# Taguchi optimization and scale up of xylanase from *Bacillus licheniformis* isolated from hot water geyser

**DOI:** 10.1186/s43141-020-00084-0

**Published:** 2020-10-22

**Authors:** Girisha Malhotra, Shilpa S. Chapadgaonkar

**Affiliations:** Department of Biotechnology, Manav Rachna International Institute of Research and Studies, Faridabad, Haryana India

**Keywords:** Xylanase, Feed enzyme, Taguchi design, Bacillus, Xylan

## Abstract

**Background:**

Xylanase is one of the widely applied industrial enzymes with diverse applications. Thermostability and alkali tolerance are the two most desirable qualities for industrial applications of xylanase. In this paper, we reveal the statistical Taguchi optimization strategy for maximization of xylanase production. The important process parameters pH, temperature, concentration of wheat bran, and concentration of yeast extract were optimized using the Taguchi L_8_ orthogonal array where the 4 factors were considered at 2 levels (high and low).

**Results:**

The optimized conditions given by model were obtained as follows: (i) pH 6, (ii) culture temperature 35 °C, (iii) concentration of xylan 2% w/v, (iv) concentration of wheat bran 2.5% w/v. The production was scaled upto 2.5 L bioreactor using optimized process parameters. A high xylanase titer of 400 U/ml could be achieved in less than 60 h of culture in the reactor.

**Conclusion:**

Optimization was successful in achieving about threefold increase in the yield of xylanase. The optimized conditions resulted in a successful scale up and enhancement of xylanase production.

## Background

Xylanase is widely used industrial enzyme, and the development of indigenous technologies is essential to improve the economy of the process applications. Microbial medium optimization is known to improve the bioprocess production yields by many folds [1, 5, 10, 16, 19]. The traditional one factor at a time (OFAT) medium optimization methodology requires too many optimization experiments and takes an unreasonable amount of time. The full factorial statistical optimization designs consider all the possible combinations of factors; a full factorial experimental design for “*n*” factors to be tested at “*k*” different levels needs “*n*^*k*^” experiments. Traditional process requires huge number of trial experiments and is tedious and time consuming. Moreover, since each factor is taken “one-at-a-time” interaction between the factors cannot be studied. In contrast, statistical optimization techniques like the Taguchi design can achieve optimization in smaller number of experiments. Optimization of four process parameters at two different levels would require 4^2^, i.e., 16 different experimental trials using the traditional one factor at a time full factorial design, whereas this optimization can be achieved in only 8 experimental trials with the Taguchi approach. Moreover, since the variables are optimized simultaneously, interaction between the factors, if any, can be identified. The Taguchi design considers two different types of factors: control factors (the factors that can be controlled) and noise factors (i.e., factors that cannot be controlled). The Taguchi method seeks to minimize the variation due to noise and to study the effect of independent variables. The orthogonal Taguchi design has been used extensively to optimize the process parameters for production of many enzymes including xylanases [6], tannase [13], alkaline protease [17], α-amylase [21], and L-asparaginase [2].

In the present study, optimization of bioprocess for thermo-alkali stable xylanase production from a newly discovered *Bacillus licheniformis* isolate from the hot water geyser in Sohna, Haryana, India, was carried out with the objective of maximization of xylanase production. This isolate has been shown to possess high xylanolytic activity in our studies [15]. Optimization of four independent parameters with an objective to maximize xylanase production was carried out at two different levels.

## Methods

### Microorganism and culture maintenance

Prior to main trials for the optimization of the medium, the isolate B2, a bacterial strain with high xylanolytic potential was selected and was identified as a strain of *Bacillus licheniformis* [15]. It was maintained in nutrient agar medium at pH 7.0 and temperature 35 °C. The glycerol stock cultures and working stock cultures were maintained as slants and revived regularly.

### Preparation of inoculum

The seed culture was prepared by inoculating actively growing culture of the *Bacillus licheniformis* strain to 50 ml of nutrient broth supplemented with 1% w/v xylan and pH 6.5 in a 250-ml Erlenmeyer flask. The culture was incubated at 35 °C with continuous shaking at 150 RPM.

### Culture medium

The basal medium used consisted of 0.5% w/v yeast extract, 0.25% w/v NaCl, 0.5% w/v NH_4_Cl, 0.025% w/v MgSO_4_7H_2_O, and 1% w/v of agricultural waste as the source of carbon in the medium. Available agro-residues: xylan, wheat bran, and sorghum stalk were procured locally. They were subjected to pretreatment, which lead to the softening and increased the accessibility of the complex carbon source. The pretreatment process involved cleaning, grinding for size reduction, sieving, and autoclaving with other medium components.

The trials for the selection of nitrogen source involved varying the type of nitrogen source (peptone, tryptone, and yeast extract and ammonium sulfate) and comparing the xylanase production in the medium. The basal medium used here was 0.5% w/v of nitrogen source, 0.25% w/v NaCl, 0.5% w/v NH_4_Cl, 0.025% w/v MgSO_4_7H_2_O, and 1% w/v wheat bran with initial medium of 7.0. During these trials, all other factors were kept constant.

The medium was autoclaved at 121 °C for 20 min. Fifty milliliter of this formulated medium was taken in 250 ml shake flask and inoculated with actively growing culture.

#### Xylanase assay

Xylanase activity was determined by 3, 5-dinitrosalicylic acid (DNSA) method. It depends on measuring the concentration of reducing sugars liberated from xylan by the activity of xylanase [3]. In brief, the culture broth was first centrifuged at 10,000 RPM for 10 min at 10 °C. One percent w/v beechwood xylan (Himedia MB141-10G) prepared in 0.05 M sodium citrate buffer pH 6.5 was used as substrate. One milliliter of substrate was incubated with 500 μl of crude enzyme (supernatant obtained from centrifuged culture broth) at 50 °C for 15 min. The reaction was terminated by the addition of 3 ml of DNSA reagent and the mixture was boiled for 10 min in a water bath. The absorbance was measured at 540 nm; after cooling, the mixture which is a measure of reducing sugar liberated due to enzyme action. A calibration curve using xylose was determined with xylose as standard. One unit of xylanase activity (U) is defined as the amount of enzyme that liberates 1 μmol of reducing sugar—xylose per min under the standard assay conditions.

$$ Xylanase\ activity\ U/ ml=\frac{Absorbance\  at\ 540\  nm\times \kern0.5em Dilution\ factor}{Time\ of\ incubation\times volume\ of\ sample\ taken} $$

#### Taguchi design for optimization of xylanase production

**Fig. 1:**
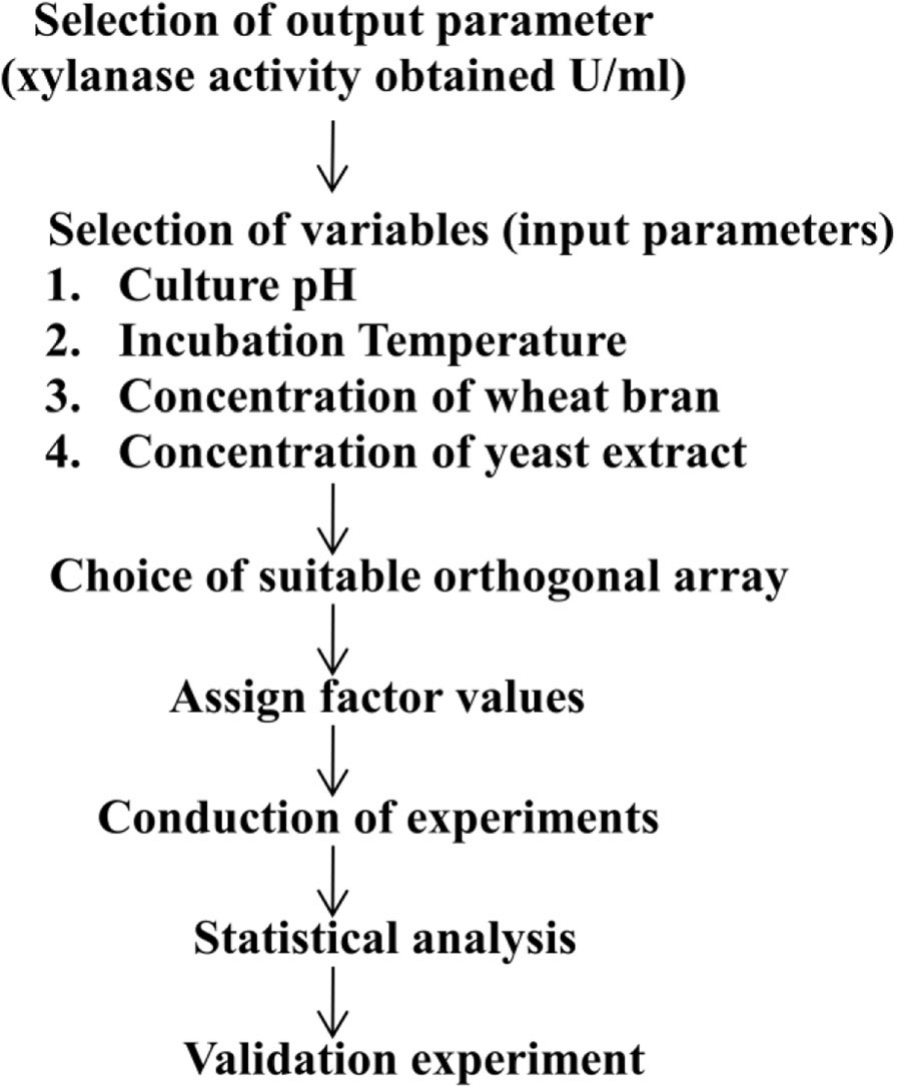
Steps in statistical optimization using the Taguchi design

The important steps in the Taguchi design have been given schematically in Fig. 1. The generation of design L8 array and analysis of results were carried out using the Minitab® (trial version) software.

The optimization was carried out using L_8_ orthogonal array where the 4 factors were considered at 2 levels (high and low). The high and low values were chosen on the basis of previous literature [9, 11, 15]. The factors considered in the optimization design were (i) concentration of wheat bran, (ii) pH (iii) culture temperature, and (iv) concentration of yeast extract. The factorial design used has been given in Table 1. During the trials, all other media components and process parameters were kept constant. Xylanase activity was measured as the “output parameter.” All of the experiments were repeated three times for obtaining statistically significant results.

**Table 1:** Taguchi L8 array for 2-level 4-factors

SN	Factors with assigned levels			
	pH	Temperature (°C)	Concentration of wheat bran (% w/v)	Concentration of yeast extract (% w/v)
1	6	35	0.5	1
2	6	35	2	2.5
3	6	45	0.5	2.5
4	6	45	2	1
5	8	35	0.5	2.5
6	8	35	2	1
7	8	45	0.5	1
8	8	45	2	2.5

### Taguchi design analysis

#### Signal to noise ratio

In the Taguchi optimization, signal to noise (S/N) ratio is the ratio of desirable “signal” to the undesirable “noise” or variation. Therefore, it can be used to determine the design quality. Since our objective is to maximize the signal factor (xylanase activity). S/N ratio for “larger the better” output was used. The effect of each factor was ranked according to its significance.

This was calculated as

$$ \mathrm{S}/\mathrm{N}=-10\ast \log \left(\sum \left(1/{\mathrm{Y}}^2\right)/\mathrm{n}\right) $$

where

S/N is the signal to noise ratio,

*Y* is the signal factor (xylanase activity),

*n* is the number of repetitions in the experiment.

#### Main effects

The main effect of each factor was calculated using the following equations.

$$ {\displaystyle \begin{array}{l}X1\ (A)=\left(a+b+c+d\right)/4=\mathrm{ME}1(A)\\ {}X2\ (A)=\left(p+q+r+s\right)/4=\mathrm{ME}2(A)\end{array}} $$

Where, *X*1 (*A*) was the mean of the output value (xylanase activity) when the factor A was at level 1 and *X*2 (*A*) was the mean of the factor A was at level 2. The mean value of *X*1 (*A*) and X2 (*A*) in different runs was computed. Thus if a, b, c, d, p, q, r, s are the output (xylanase activities) obtained in different runs where the factor A was at level 1 or level 2. The delta value of difference in the effects is obtained as

$$ \Delta =\mathrm{X}1\ (A)-\mathrm{X}2(A) $$

The importance of each factor on xylanase activity was determined by its rank which is assigned on the basis of the relative delta value.

#### Analysis of variance or ANOVA

ANOVA was applied to determine the statistical significance of the results. The variance due to the factors or their combinations as well as the variation due to noise was determined. The test data points were used to calculate the total variance of the desired output. The measure of “relative significance” was based on an *F* test. The factors having *p* < 0.05 were identified as significant. After determination of optimal process parameters, validation experiments were carried out and the xylanase activity obtained in the validation experiments were compared with the values predicted by the model.

#### Bioreactor studies

The xylanase production was scaled up to laboratory–scale 5 L bioreactor (Bioage, India) with working volume of 4 L. The bioreactor possessed the automated systems to control and monitor dissolved the oxygen (DO), pH, agitator, temperature, and foaming. The pH electrode was first calibrated using the standard buffers of pH 7.0 and 9.0. The DO probe was calibrated with sodium sulfite (1 N) and then saturated with oxygen to calibrate the range 0 to 100% respectively. The process parameters that were optimized in the shake-flask cultures were used for the bioreactor studies. The medium consisted of 2%w/v wheat bran, 2.5% w/v yeast extract, 0.25% w/v NaCl, 0.5% w/v NH_4_Cl, and 0.025% w/v MgSO_4_7H_2_O. The fermenter vessel along with the formulated medium was sterilized at 121 °C at 15 psi for 30 min. Once the medium cooled to the process temperature, it was inoculated with actively growing culture of *Bacillus licheniformis* isolate (10%v/v). The process parameters were controlled at pH (6.0) and the culture temperature was maintained at 35 °C and dissolved oxygen at 60% during the reactor run. The samples were withdrawn at 12 h intervals and analyzed for bacterial growth and xylanase activity.

## Results

### Selection of carbon and nitrogen source

**Fig. 2:**
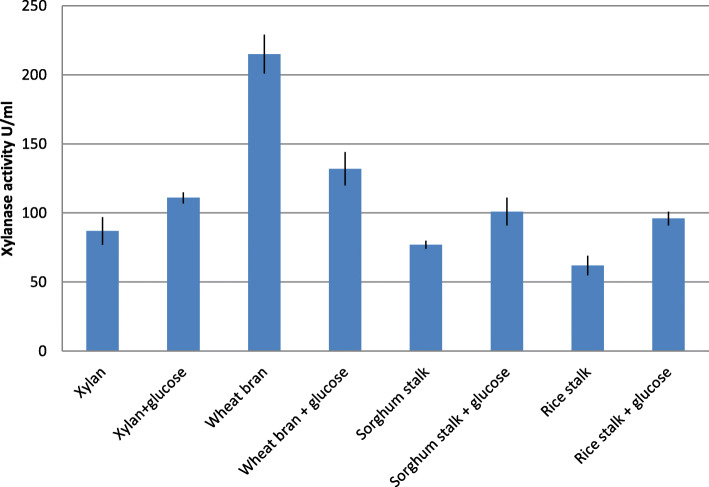
Effect of different carbon sources on xylanase production

The xylanase yield obtained with the preliminary experiments conducted for the selection of carbon sources has been given in Fig. 2. In these runs, the composition of the basal medium was kept constant while only the type of agro-residue was varied. The concentration of agro-residue carbon source was fixed at 1% w/v. The xylanase activity obtained after 72 h of culture was compared (Fig. 2).

**Fig. 3:**
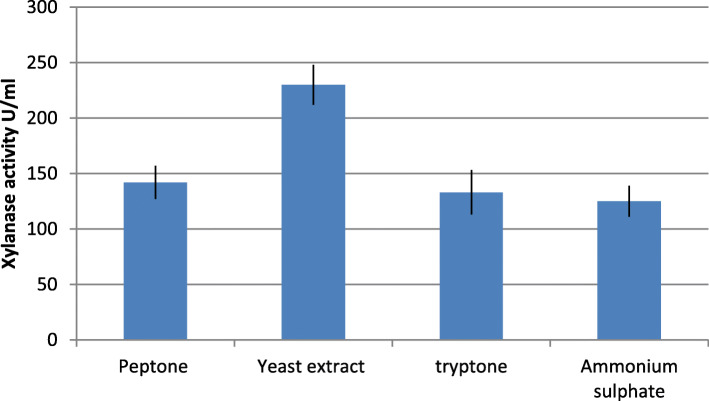
Effect of different nitrogen sources on xylanase production

It can be seen that the medium containing wheat bran as the sole carbon source showed highest xylanase production. Therefore, wheat bran was selected as the component of xylanase production medium. The substrates when formulated with glucose did not improve xylanase production to a significant extent. The xylanase yield obtained with the experiments conducted for the selection of nitrogen sources has been given in Fig. 3. Maximum xylanase production was obtained with yeast extract and hence it was chosen as the nitrogen source in the xylanase production medium.

**Fig. 4:**
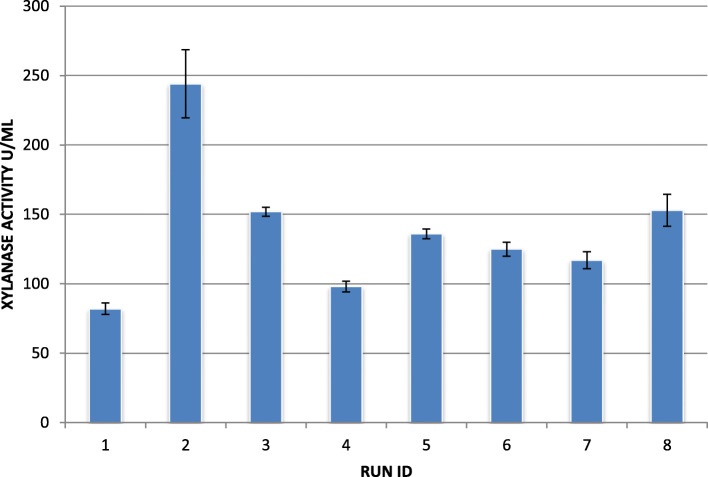
Xylanase activity obtained in trial runs

The Taguchi optimization trials were conducted as per the design (Table 1) and xylanase activity for each trial run was determined as the output parameter (Fig. 4). The MINITAB statistical software package (Design Expert, Trial version) was used to generate and analyze the experimental design.

**Fig. 5:**
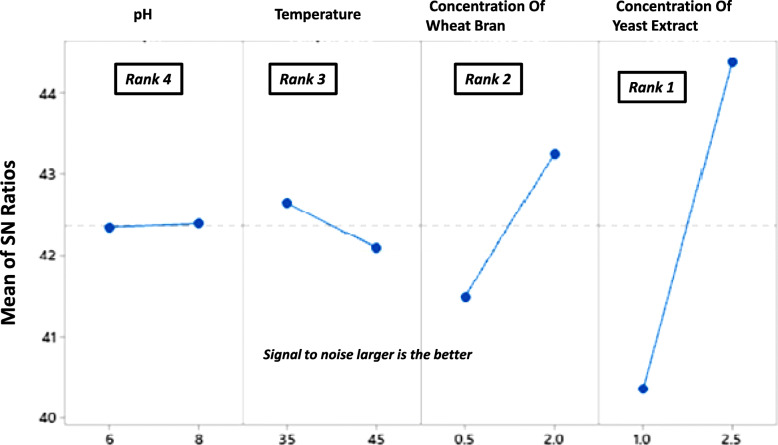
Signal-to-noise ratio computed for each factor

**Fig. 6:**
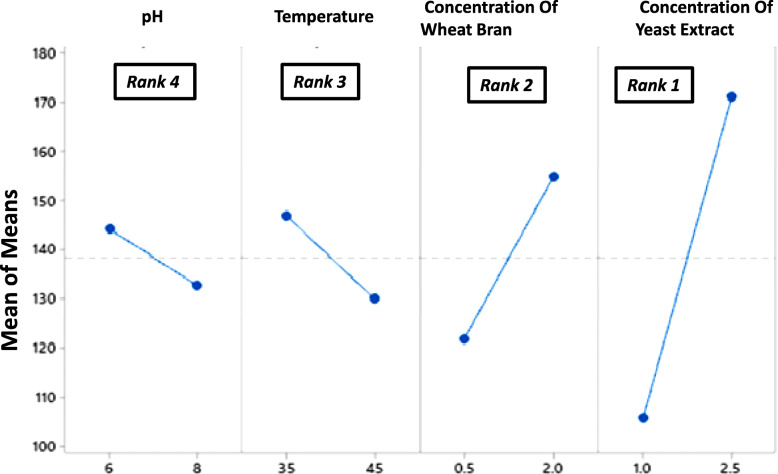
Main effects plot for data means obtained in the optimization trial runs

It is evident that the run 2 with factors setting at pH (6.0), temperature (35 °C), wheat bran (2% w/v), and yeast extract at 2.5% (w/v) gave the highest xylanase production at 244 U/ml. Whereas, the trial run 1 conducted with pH (6.0), temperature (35 °C), wheat bran (0.5% w/v), and yeast extract at 1% (w/v) resulted in the lowest yield of xylanase (82 U/ml). The large effect of the process parameters on the xylanase yield is thus evident from the results. Figures 5 and 6 illustrate for factor-wise S/N ratio and the main effects plots.

It is evident from the S/N and the main effects plots that the concentration of yeast extract had the greatest effect on xylanase production followed by wheat bran concentration, culture temperature, and medium pH. The factor yeast extract concentration with greatest delta (∇) value was assigned rank 1 depicting its highest effect on xylanase. Similarly, concentrations of wheat bran and incubation temperature rank second and third respectively. The initial media pH had the least effect on xylanase production and was assigned rank 4. In order to determine the statistical significance of each factor on xylanase production, analysis of variance (ANOVA) was carried out (Table 2). It was observed that the factors pH and temperature did not contribute significantly to xylanase production. In order to increase the accuracy of the model the effects of these were pooled (Table 3).

**Table 2:** ANOVA table for means

Source	DF	Seq SS	Adj SS	Adj MS	***F***	***P***
pH	1	258.0	258.0	258.0	0.14	0.729
Temperature	1	580.5	580.5	580.5	0.33	0.608
Wheat bran	1	2189.4	2189.4	2189.4	1.23	0.349
Yeast extract	1	8562.7	8562.7	8562.7	4.80	0.116
Residual error	3	5349.1	5349.1	1783.0		
Total	7	16,939.7				
Wheat bran	1	2189	2189	2189	1.77	0.241
Yeast extract	1	8563	8563	8563	6.92	0.047
Residual error	5	6188	6188	1238		
Total	7	16,940				

Linear regression model was used to fit the experimental data and to identify the relevant model terms. The regression equation obtained was

Xylanase concentration = 138.40 − 16.54 × wheat bran concentration − 32.72 × yeast extract concentration

Finally, the optimized settings for each parameter were obtained as given in the Table 3.

**Table 3:** Optimized settings for each factor

pH	Temperature (°C)	Concentration of wheat bran (% w/v)	Concentration of yeast extract (% w/v)
6	35 °C	2	2.5

The validation experiments were carried out according to the following factor settings as given in Table 4.

**Table 4:** Factor settings for validation experiment and the predicted and actual xylanase activity

SN	Concentration of wheat bran (% w/v)	Concentration of yeast extract (% w/v)	Predicted xylanase activity by the model	Xylanase activity obtained
1	0.5	1	97.41	89
2	1	2	220.38	202
3	2	2.5	253. 28	244

It can be observed from Table 4 that the model could accurately predict the xylanase production at different settings of the factors and hence it could be validated.

### Bioreactor studies

**Fig. 7:**
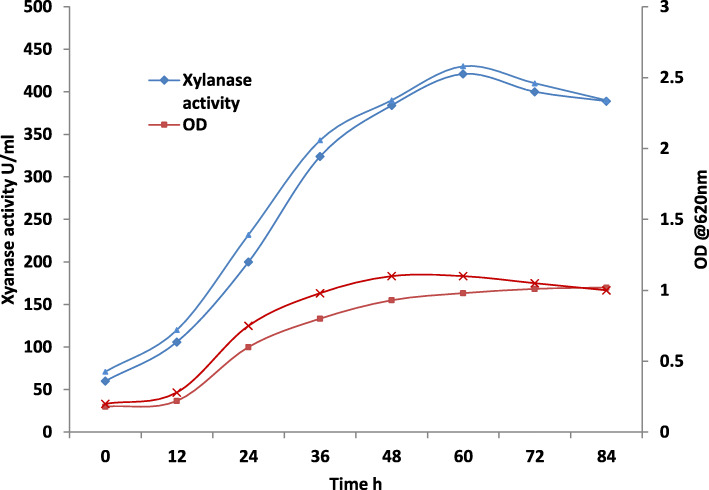
Xylanase production and growth obtained in stirred tank reactor

**T**he bioreactor was operated using standard operating procedure as mentioned in the “Methods” section. The process parameters were controlled at optimized values of pH 6.0 and temperature 35 °C. The agitator was operated at 150 RPM and DO was controlled at 60%. The xylanase activity and optical density at 620 nm was measure during the reactor run. Figure 7 gives the time course of growth and xylanase production in the batch stirred tank reactor for two different reactor runs.

It could be observed that peak xylanase activity was achieved in 60 h in the bioreactor which is much faster as compared to the shake-flask experiments where the peak activity is obtained in 72 h [15]. Moreover, the enzyme concentration obtained was much higher than the shake flask cultures.

## Discussion

Every organism has unique medium requirements that would direct its cellular machinery toward the production of the desired compound. The type and concentration of media components and process parameters such as the rate of agitation, pH, and culture temperature have a significant effect on bioprocess yields. The concentration of carbon and nitrogen are often found to be limiting in the bioprocesses due to their higher consumption rates. It has been elucidated by several studies that bacterial xylanase production is repressed in the presence of easily available carbon sources such as glucose due to carbon catabolite repression and induced by the availability of xylan (xylooligosaccharides) [12]. Irfan et al. [9] carried out optimization of process parameters for xylanase from an isolate of *Bacillus* sp. They concluded that pH (range tested 4-10), temperature (25-50 °C), substrate concentration (0.5 to 3% w/v), and inoculum size (0.5 to 3% v/v) affected xylanase production significantly. The optimum parameter levels were found to be pH 8, temperature 35-40 °C (for *Bacillus subtilis* and *Bacillus licheniformis* strains) 2% w/v and inoculum size 2-3% v/v. Statistical optimization of xylanase production from *Bacillus tequilensis* strain using response surface methodology was carried out by Khusro et al. [11]. They reported the optimized component levels as birchwood xylan (1.5% w/v), yeast extract (1% w/v), incubation temperature (40 °C), and time period (24 h). Shanthi and Royman [20] attempted the optimization of process parameters for xylanase production by two strains of *Bacillus* and reported that the maximum xylanase levels were obtained at pH 9.0, 55 °C for *Bacillus* sp. MCC2728 and 50 °C for *Bacillus* sp*.* MCC2727, 5% v/v inoculum, and agitation speed (150 rpm). Yeast extract and peptone were reported to be the best nitrogen sources and wheat bran was found to be the best carbon source. Our previous studies on crude xylanase had showed an optima of pH 6.5 (enzyme stability range from 6-8) and temperature optima of 70 °C (stable range 40-80 °C) [15]. Based on these insights, concentrations of the carbon and nitrogen sources, initial pH of the culture medium, and culture temperature were chosen to be optimized for the maximization of xylanase production.

Locally available agro-residues such as xylan, wheat-bran, sorghum stalk, and rice stalk were used. These residues provide xylan for the induction of xylanase production. The selection of wheat-bran as the carbon source and yeast extract as the nitrogen source was made on the basis of comparison of xylanase production obtained with varying the type of agro-residue and nitrogen sources only (Figs. 2 and 3).

The utility of the Taguchi robust design for optimization of process parameters is well-known as shown by several recent reports of numerous processes that have been optimized using this design ([4, 7], and [18]), and the results from the present study also consolidate the applicability of the strategy. The Taguchi design was applied and analyzed, the optimized conditions given by the model were as follows: (i) pH 6, (ii) culture temperature 35 °C, (iii) concentration of xylan 2% w/v, and (iv) concentration of wheat bran 2.5% w/v. The model developed for the relation of factor levels to xylanase could efficiently predict the xylanase production as given by the results of the validation runs. The study revealed that xylanase production depended on the concentration of yeast extract and xylan whereas pH and culture temperatures did not affect the production to significant extent in the particular experimental range. Kumar et al. [14] also found the influence of wheat bran and yeast extract concentration on xylanase production by alkaliphilic *Bacillus pumilus* strain. The strategy exemplifies an efficient method for reducing the time required for upstream process optimization. Higher xylanase yield and reduction of batch time was achieved in the reactor. This is likely due to the effective control of bioprocess parameters in the reactor as compared to the shake flask conditions. Peak growth was achieved at 48 h of culture in the reactor, whereas the peak xylanase activity was obtained in 60 h of reactor run. In the future work, production process would be optimized exclusively using bioreactor keeping in view the other important control parameters such as DO, rate of agitation, and antifoaming agent. Xylanase activity fell toward the end of the reactor run which is likely due to the proteolytic degradation. Holland et al. [8] reported the degradation of xylanases by extracellular proteases and recommend use of protease inhibitors in the medium.

## Conclusion

The present paper gives an effective and simple strategy for the process optimization of xylanase production from a newly isolated *Bacillus licheniformis* strain with promising xylanolytic potential. Medium optimization was successful in achieving more than threefold increase in xylanase production using optimized conditions. The production was scaled up to 2.5 L working volume lab scale reactor, employing the parameters as optimized in the shake flask experiments. A high xylanase titer of 400 U/ml could be achieved in less than 60 h of culture in the reactor. This paper describes a simple and time efficient strategy to bring about fold increase in xylanase production by the optimization of process parameters.

## Data Availability

The datasets used and/or analyzed during the current study are available from the corresponding author on reasonable request.

## Abbreviations

OFAT: One factor at a time
DNSA: 3, 5-dinitrosalicylic acid
S/N ratio: Signal to noise ratio
ANOVA: Analysis of variance
Coef: Coefficient
SE Coeff: Standard error of the coefficient
T: Test statistic for Student’s *t* distribution
*P*: *P* value
DF: Degree of freedom
Seq SS: Sequential sums of squares
Adj SS: Adjusted sum of squares
Adj MS: Adjusted mean squares
